# No evidence in support of arthroscopic partial meniscectomy in adults with degenerative and nonobstructive meniscal symptoms: a level I evidence-based systematic review

**DOI:** 10.1007/s00167-022-07040-0

**Published:** 2022-07-01

**Authors:** Filippo Migliorini, Francesco Oliva, Jörg Eschweiler, Francesco Cuozzo, Frank Hildebrand, Nicola Maffulli

**Affiliations:** 1 Department of Orthopaedic, Trauma, and Reconstructive Surgery, RWTH University Hospital, Pauwelsstraße 30, 52074 Aachen, Germany; 2 Department of Medicine, Surgery and Dentistry, University of Salerno, 84081 Baronissi, SA Italy; 3 School of Pharmacy and Bioengineering, Faculty of Medicine, Keele University, Stoke-on-Trent, ST4 7QB England; 4 Barts and the London School of Medicine and Dentistry, Centre for Sports and Exercise Medicine, Mile End Hospital, Queen Mary University of London, E1 4DG London, England

**Keywords:** Arthroscopy, Partial meniscectomy, Meniscus, Physical therapy

## Abstract

**Purpose:**

It is unclear whether the results of arthroscopic partial meniscectomy (APM) are comparable to a structured physical therapy (PT). This systematic review investigated efficacy of APM in the management of symptomatic meniscal damages in middle aged patients. Current available randomised controlled trials (RCTs) which compared APM performed in isolation or combined with physical therapy versus sham arthroscopy or isolated physical therapy were considered in the present systematic review.

**Methods:**

This systematic review was conducted according to the 2020 PRISMA statement. All the level I RCTs which investigated the efficacy of AMP were accessed. Studies which included elderlies with severe OA were not eligible, nor were those in which APM was combined with other surgical intervention or in patients with unstable knee or with ligaments insufficiency. The risk of bias was assessed using the software Review Manager 5.3 (The Nordic Cochrane Collaboration, Copenhagen). To rate the quality of evidence of collected outcomes, the Grading of Recommendations, Assessment, Development, and Evaluation (GRADE) was used.

**Results:**

Data from 17 studies (2037 patients) were collected. 48.5% (988 of 2037 patients) were women. The mean age of the patients was 52.7 ± 3.9 years, the mean BMI 27.0 ± 1.3 kg/m^2^. The current evidence suggests no difference in functional PROMs (quality of the evidence: high), clinical PROMs (quality of the evidence: high), pain (quality of the evidence: high), quality of life (quality of the evidence: high), physical performance measures (quality of the evidence: moderate), and OA progression (quality of the evidence: moderate).

**Conclusions:**

The benefits of APM in adults with degenerative and nonobstructive meniscal symptoms are limited. The current evidence reports similarity in the outcome between APM and PT. Further long-term RCTs are required to investigate whether APM and PT produce comparable results using validated and reliable PROMs. Moreover, future RCTs should investigate whether patients who might benefit from APM exist, clarifying proper indications and outcomes. High quality investigations are strongly required to establish the optimal PT regimes.

**Level of evidence:**

Level I.

## Introduction

Degenerative meniscal damage is common in adults [14, 27]. At present, the optimal management for non-traumatic, degenerative meniscal damage remains controversial [8, 38]. Meniscal tears are defined as intrameniscal linear signal penetrating one or both surfaces of the meniscus at magnetic resonance imaging (MRI) [10]. Arthroscopic partial meniscectomy (APM) has been advocated to manage degenerative and obstructive (i.e., inducing locking of the knee joint) meniscal damage [8, 22]. However, in adults with degenerative and nonobstructive meniscal symptoms the superiority of APM over a well-structured physical therapy (PT) programme is debated [1, 9, 19, 35, 44, 47, 54]. Previous systematic reviews were inconsistent and found none to slightly better outcome in APM compared to PT [1, 9, 19, 33, 35, 44, 47, 54, 57, 59]. However, additional long-term randomised controlled trials (RCTs) [4, 6, 40, 42] and additional follow-up of previously published pivotal RCTs [31, 51], which have not yet been included in any previous systematic review, have been recently published. Therefore, an update of the clinical evidence is necessary.

This systematic review investigated the efficacy of APM in the management of symptomatic meniscal damage in middle aged patients. Current available RCTs which compared APM performed in isolation or combined with PT versus sham APM or isolated PT were considered in the present investigation. It was hypothesized that AMP is not superior compared to PT or sham APM in patient reported outcome measures (PROMs), physical performance measures, and progression of osteoarthritis (OA).

## Materials and methods

### Eligibility criteria

All the clinical trials which investigated the efficacy of AMP were accessed. Given the authors language capability, articles in English, German, Italian, French, and Spanish were eligible. Only RCTs with level I of evidence, according to Oxford Centre of Evidence-Based Medicine [25], were considered. Animal, biomechanics, and computational studies were not considered. Reviews, comments, editorials, and expert opinion were not eligible. Studies which included patients with severe OA (Kellgren–Lawrence IV) were not included. Studies which investigated patients with acute meniscal tears or mechanical symptoms were not eligible. Studies in patients older than 70 years were not included, nor those conducted in skeletally immature patients. Studies in which APM was combined with other surgical intervention were excluded. Studies which included patients with unstable knee or ligaments insufficiency were not eligible. Only studies which investigated patients who underwent exclusively isolated APM or APM combined with PT were included. Studies which reported data on patients who received meniscal allografts or bio-scaffolds were not considered.

### Search strategy

This systematic review was conducted according to the Preferred Reporting Items for Systematic Reviews and Meta-Analyses (PRISMA 2020 statement) [43]. The following algorithm was preliminarily set out:

- Population: adults aged 30–70.
- Problem: degenerative and nonobstructive meniscal damage.
- Intervention: APM in isolation or combined to PT.
- Comparison: PT, sham arthroscopy.
- Outcomes: PROMs, physical performance measures, OA progression.
- Study design: RCT.
- Duration: minimum 3 month follow-up.

In March 2022, the following databases were accessed: Pubmed, Web of Science, Google Scholar, Embase. No time constrains were used for the search. The following keywords were used in combination using the Boolean operators: ((knee OR meniscus OR meniscal OR meniscopathy) AND (damage OR injury OR tear OR pain) AND/OR (symptoms OR overuse OR degenerative OR nonobstructive OR mechanical OR locking)) AND ((arthroscopy OR arthroscopic) AND partial meniscectomy) AND/OR (physical AND/OR therapy OR exercises OR training OR physiotherapy OR rehabilitation)) AND (outcome OR return OR patient reported outcome measures OR proms OR vas OR visual analog scale OR womac OR Western ontario and mcmaster universities osteoarthritis index OR koos OR knee injury and osteoarthritis outcome score OR osteoarthrosis OR Kellgren–Lawrence OR performances).

### Selection and data collection

Two authors (F.M and F.C.) independently performed the database search. All the resulting titles were screened, and if suitable, the abstract was accessed. The full-text of the abstracts which matched the topic were accessed. The bibliography of the full-text articles was also screened by hand for inclusion. Any disagreements were discussed and settled by a third author (**).

### Data items

Two authors (F.M. and F.C.) independently performed data extraction. Study generalities were collected: author, year, level of evidence, study design, length of the follow-up, type of treatment, physical therapy or home exercise protocol, number of patients and related mean age and body mass index (BMI). At each follow-up, data concerning PROMs, physical performance measures, and progression of OA were collected.

### Study risk of bias assessment

Two reviewers (F.M. and F.C.) independently evaluated the risk of bias of the extracted studies according to the Cochrane Handbook for Systematic Reviews of Interventions guidelines [11]. The risk of bias was assessed using the software Review Manager 5.3 (The Nordic Cochrane Collaboration, Copenhagen). The following endpoints were evaluated: selection, detection, performance, attrition, reporting, and other bias. The selection bias rates the method used to generate the allocation sequence. The detection and performance biases investigate the quality of blinding methods of assessor and patients, respectively. Attrition bias refers to the quality of outcome data for each study endpoint, evaluating attrition and exclusions in the study. Reporting bias explores the possibility of selective outcome reporting by the authors.

### Quality of recommendations

To rate the quality of evidence of collective outcomes the Grading of Recommendations, Assessment, Development, and Evaluation (GRADE) system was used [2, 21]. The GRADE was performed by two authors (F.M. and F.C.). The GRADE is a sensitive and transparent tool to rate the quality of the recommendations which arise from the included studies, assessing the reliability of scientific evidence and formulating evidence-based clinical recommendations.

### Statistical analyses

The statistical analyses were performed using the software IBM SPSS version 25. For continuous variable, the arithmetic mean and standard deviation was used.

## Results

### Study selection

**Fig. 1:**
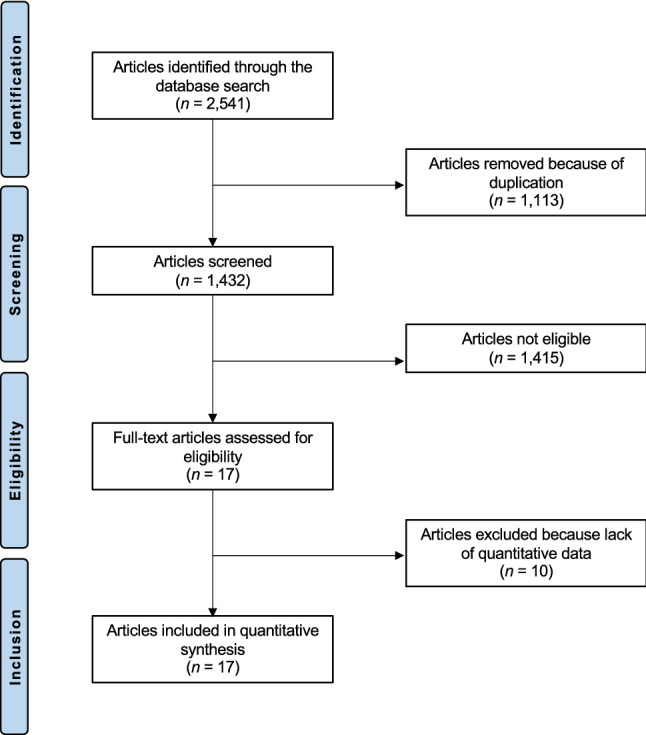
Flow chart of the literature search

A total of 2545 studies resulted from the databases search. Of them, 1113 were excluded as they were duplicates. A further of 1415 studies were excluded with reason: not focusing on APM (*N* = 523), combining APM with other surgical interventions (*N* = 78), investigating APM in the elderlies or young population and/or in advanced OA (*N* = 89), not reporting the clinical or imaging outcomes of APM (*N* = 28), including patients with mechanical symptoms (*N* = 8), study design (*N* = 684), language limitations (*N* = 5). Finally, 17 RCTs were included in the present systematic review. The flow chart of the literature search is shown in Fig. 1.

### Study risk of bias assessment

**Fig. 2:**
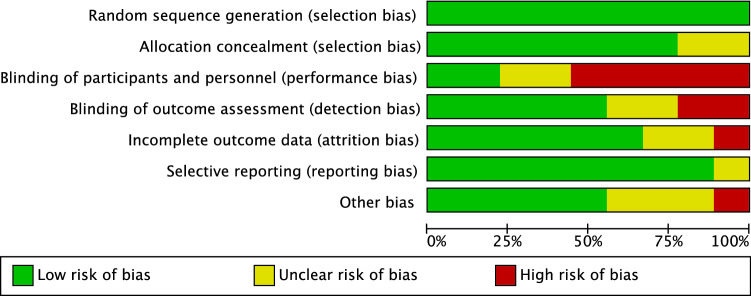
Cochrane risk of bias tool

Given the high quality of randomisation and allocation concealment in most studies, the overall risk of selection bias was low. Performance bias was moderate, as only four RCTs (two authors), which evaluated the efficacy of sham arthroscopy versus APM, were conducted in a double blinded fashion. Detection bias was low to moderate, since few studies did not conduct assessors blinding. Detection and attrition biases were low, the risk of other biases was low to moderate. Concluding, the overall risk of publication bias was low, attesting to the present systematic review a good quality of the methodological quality assessment (Fig. 2).

### Study characteristics and results of individual studies

Data from 2,037 patients were collected. 48.5% (988 of 2037 patients) were women. The mean age of the patients was 52.7 ± 3.9 years, and the mean BMI 27.0 ± 1.3 kg/m^2^. Table 1 shows the generalities, patient demographic, and the type of PROMs referred in the included studies.

**Table 1:** Generalities, patient demographic, and the PROMs used in the included studies (EQ5D: EuroQol; IKDC: International Knee Document Committee; HAD: Hospital Anxiety and Depression Scale; VAS: Visual Analog Scale; WOMAC: Western Ontario and McMaster Universities Osteoarthritis Index; KOOS: Knee injury and Osteoarthritis Outcome Score subscale; PSFS: Patient-Specific Functional Scale; SF36: Short Form 36; GPE: Global Perceived Effect; WOMET: Western Ontario Meniscal Evaluation Tool)

Author, year	Follow-up (months)	Degree of OA	PROMs	Treatment	Patients (*n*)	Mean age	Female (*n*)	Mean BMI
Basar et al. 2021 [4]	2, 6	KL 0 to III	WOMAC, VAS, ROM	APM (exercise at home/6–8 weeks)	41	48.4	26	27.5
				PT (3 sessions week/4 weeks)	44	50.9	29	28.7
Berg et al. 2020 [6]	3, 12, 24, 60	KL 0 to I	KOOS	APM (exercise at home/12 weeks)	70	48.9	27	26.0
				PT (3 sessions week/12 weeks)	70	50.2	27	26.5
Gauffin et al. 2014 [18]	3, 12	KL 0 to I	KOOS, EQ5D, VAS	APM and PT	75	54.0	22	
				PT (2 session week/12 weeks)	75	54.0	19	
Herrlin et al. 2007, 2013 [22, 23]	2, 6, 12, 24, 60	Ahlbäck 0 to I	KOOS, Lysholm, Tegner, VAS	APM and PT	47	54.0	19	25.7
				PT (2 session week/8 weeks)	49	56.0	16	25.9
Katz et al. 2013, 2020 [30, 31]	3, 6, 12, 60	KL 0 to II	KOOS, WOMAC	APM and PT	174	58.6	99	30.2
				PT (2 session week/6 weeks)	177	57.2	102	30.2
Kise et al. 2016 [34, 60]	3, 12, 24	KL 0 to II	KOOS	APM (exercise at home/12 weeks)	70	48.9	43	26.0
				PT (2–3 session week/12 weeks	70	50.2	43	26.4
Noorduyn et al. 2020 [40]	3, 6, 12, 24	KL 0 to III	PSFS	APM (exercise at home/8 weeks)	159	57.6	81	26.7
				PT (2 session week/8 weeks)	162	57.3	82	27.2
Osteras et al. 2021 [42]	3	KL 0 to II	VAS, KOOS, HAD	APM	8	52.7	3	
				PT (3 times/week for 12 weeks)	9	47.0	1	
Roos et al. 2018 [50]	24	KL 0 to II	KOOS, EQ5D, VAS, SF36, GPE	APM	44	47.2	9	27.6
				Sham Arthroscopy	44	47.4	12	26.0
Sihvonen et al. 2013, 2018, 2020 [51–53]	12, 24, 60	KL 0 to I	Lysholm, WOMET, VAS	APM	70	52.7	28	26.9
				Sham Arthroscopy	76	52.7	29	27.9
Stensrud et al. 2015 [56]	3	KL 0 to II		APM and PT	42	48.6	16	26.3
				PT (2 session week/12 weeks)	40	49.2	13	26.9
Van de Graaf et al. 2018 [58]	3, 6, 12, 24	KL 0 to III	IKDC, VAS, RAND-36, Tegner	APM (exercise at home/6 weeks)	158	57.6	80	26.7
				PT (2 session week/6 weeks)	161	57.3	81	27.2
Yim et al. 2013 [60]	3, 6, 12, 24	KL 0 to I	VAS, Lysholm, Tegner	APM	50	54.9	41	25.0
				PT (3 session week/3 weeks)	52	57.6	40	26.4

### Quality of recommendations

**Fig. 3:**
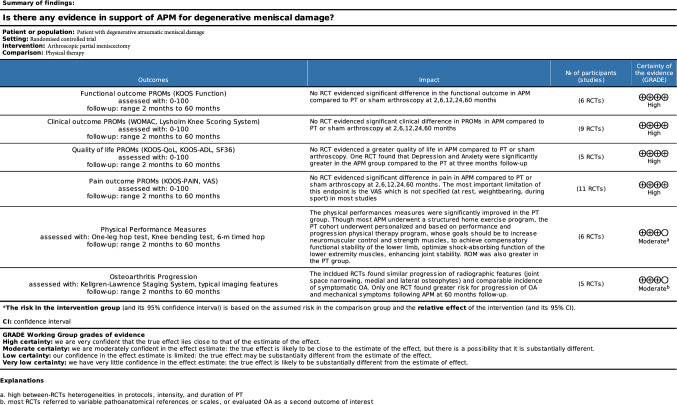
GRADE

The GRADE found an overall high quality of recommendations. PROMs which evaluated the quality of life, pain, and the clinical and the functional outcome were clearly reported, unbiased, and with minimal inconsistencies. Heterogeneities were found in physical performance measures and OA progression. Concluding, the GRADE results in a moderate to high certainly of the evidence (Fig. 3).

## Discussion

**Fig. 4:**
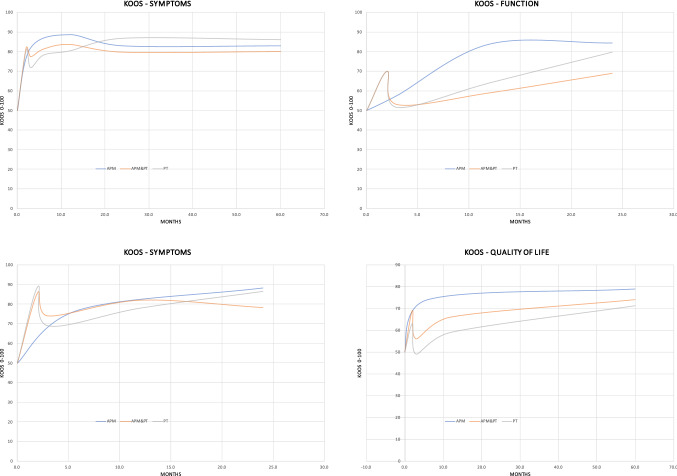
Trend of the KOOS in the included studies

**Fig. 5:**
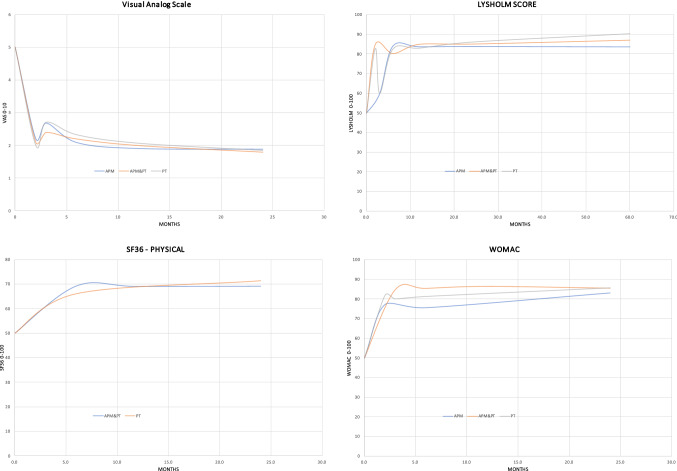
Trend of the PROMs in the included studies

According to the main findings of the present systematic review, there is no evidence in support of APM in adults with non-traumatic and nonobstructive meniscal damages. No difference was found in PROMs (Figs. 2, 3) and progression of OA between APM and PT. Physical performance measures, as expected, were worse in APM compared to PT. Adults with non-traumatic and degenerative meniscal damage could benefit from a personalized and performance and progression-based physical therapy program, whose goals should be to increase neuromuscular control and muscles strength, to achieve compensatory functional stability of the lower limb, optimize shock-absorbing function of the lower extremity muscles, and enhancing joint stability (Figs. 4, 5).

Similar to the present study, several previous systematic reviews found no evidence in support of APM over the PT [1, 9, 19, 47, 54]. On the contrary, Van de Graaf et al. [59] in a systematic review of five RCTs found that APM yielded better functional outcomes and pain control at 3–6 months, whereas from 12 to 24 months the results were comparable. Li et al. [35] systematically reviewed six RCTs showing better results from APM up to 12 months, and no difference after 24 months. Pan et al. [44] conducted a systematic review on six RCTs comparing APM combined with PT versus PT in isolation. APM combined with PT was more effective to improve range of motion and pain control in the early postoperative period.

Two RCTs investigated APM versus sham APM [50–53]. Overall, these RCTs found that isolated APM versus sham APM provided comparable results [50–53]. Sihvonen et al. [51–53] compared 146 patients undergoing APM versus sham APM. At 12 and 24 month follow-up no difference was found in Lysholm and Western Ontario Meniscal Evaluation Tool (WOMET), and in VAS after training [52, 53]. At 60 month follow-up, there was a consistent, slightly greater risk for progression of OA and mechanical symptoms in the APM group, although no relevant between-group differences in PROMs were reported [51]. Similar entry criteria were used by Roos et al. [50], which randomly allocated 44 patients aged 35–55 years to receive APM or sham APM. At 24 month follow-up, the KOOS and all its subscales were similar between the two groups [50]. Similarity was also found in EQ5D, VAS, SF36, and Global Perceived Effect (GPE) [50]. Physical performance measures, such as the one-leg hop test (both legs), knee bending test (both legs), and the isometric knee extensor strength (both legs) were also similar between APM and sham APM [50].

Seven studies compared isolated APM versus isolated PT [4, 6, 34, 40, 42, 58, 60]. Overall, these RCTs found no clinical and imaging benefits of APM over isolated PT [4, 6, 34, 40, 42, 58, 60]. Previous evidence found that, in patients assigned to physical therapy who eventually needed surgery, the delay resulting from a trial of conservative management did not impair the outcomes at 12 months after the initial presentation [26]. Van de Graaf [58] compared APM versus PT in 321 patients aged 45–70 years. At 24 month follow-up PT was non inferior to APM for knee function in IKDC, VAS, RAND-36 Physical Component Score, Tegner Activity Scale, and progression of OA using the Kellgren–Lawrence scale. Noorduyn et al. [40] investigated the effectiveness of early APM versus PT in patients aged 45–70 years with a symptomatic meniscal tear. At 2 year follow-up, no relevant difference between the two cohorts in the Dutch version of the Patient-Specific Functional Scale (PSFS) were evidenced [40]. Kise et al. [34] compared PT versus APM in 140 adults aged 35–60 years. At 3, 12, and 24 months, no clinically relevant benefit of APM over PT was found in KOOS and adverse events [34]. As expected, muscle strength had greatly improved in the PT group at 3 months [34]. 19% (30 of 70 patients) allocated to PT underwent APM, with no additional benefit within the 2 year follow-up [34]. Basar et al. [4] randomly allocated 192 patients to APM versus PT. The authors also evaluated whether the addition of hyaluronic acid promoted additional benefit in WOMAC, VAS, and range of motion. At 2 and 6 month follow-up, no difference was found in PROMs; however, the PT group demonstrated greater range of motion. Finally, the use of hyaluronic acid did not produce any clinical benefit in either group. The inefficacy of intraarticular infiltrations in APM, such as hyaluronic acid and/or platelet rich plasma (PRP), is also supported by previous evidence [5, 12, 13, 15, 20, 28, 29, 32, 38, 45]. Berg et. [6] conducted a RCT on 140 patients aged 35–60, 96% of them without evidence of OA at imaging. At 5 year follow-up, both groups reported similar progression of radiographic features (joint space narrowing, medial and lateral osteophytes) and comparable incidence of OA [6]. No difference was found in KOOS at 5 year follow-up [6]. Moreover, no statistically significant or clinically relevant improvement of the subscales of the KOOS were found from baseline to last follow-up in both groups. Osteras et al. [42] randomly allocated 17 adults with meniscal symptoms lasting > 3 months and suitable for APM to receive PT or APM. At 3 months, there was no difference in VAS and KOOS [42]. The authors examined also the dynamic quadriceps muscle strength [24], which was similar between the groups. However, at 3 month follow-up, the PT group demonstrated less anxiety and depression according to the Hospital Anxiety and Depression Scale (HAD) [42]. Yim et al. [60] conducted an RCT comparing APM versus PT in patients with degenerative horizontal tear of the medial meniscus. The authors found no differences in VAS, Lysholm score, Tegner activity scale, and patient subjective knee pain and satisfaction at 2 year follow-up [60].

Six RCTs compared APM combined with PT versus PT in isolation [18, 22, 23, 30, 31, 55]. Herrlin et al. [22, 23] compared APM combined with PT versus PT in isolation in 90 patients. At 6, 24, and 60 month follow-up, KOOS, Lysholm, Tegner, and VAS were similar between APM combined with PT and PT alone [22, 23]. No difference in OA progression was found in both cohorts at 5 year follow-up [22, 23]. Katz et al. [30, 31] randomly assigned 351 patients older than 45 with Kellgren–Lawrence 0–II to undergo APM combined with PT or PT in isolation. At 6 and 12 months postoperatively, 30% of patients allocated to PT had decided to undergo surgery, and 6% of patients assigned to APM had decided not to undergo surgery [30]. The KOOS pain and subscale function of the WOMAC did not evidence difference between the two groups at 3, 6, 12, 24, 36, and 48 months [31]. At 60 month follow-up, 9.2% and 5.1% of patients allocated, respectively, to APM and PM underwent total knee replacement [31]. Gauffin et al. [18] randomly allocated 150 patients to APM combined with PT or isolated PT. At 3 and 12 month follow-up, the combined APM and PT group reported greater pain subscale of the KOOS. No other difference was found in EuroQol (EQ5D) [46] and VAS [18]. Stensrud et al. [55] compared APM in combination with PT versus PT in isolation. They included 82 patients with symptomatic unilateral, nontraumatic, meniscus tears, aged 35–60. At 3 months, the PT group evidenced greater quadriceps function, isokinetic knee extension and flexion peak torque [55]. No difference was found at 3 months in the maximum number of knee-bends in 30 s, the one-leg hop for distance, and the 6 min timed hop [55]. These functional tests have been validated in patients with meniscal symptoms [3, 7, 41, 49].

The results of the present study should be considered in the light of several limitations. Patients allocated to APM did not undergo an individualized and supervised rehabilitation program but a program of structured home exercises. This program was heterogeneous in content, intensity, and duration. Similar considerations are applicable to the PT group: though all the physiotherapy programmes aimed to increase neuromuscular control and muscle strength, some differences in methods, content, intensity, and duration were evident. Most studies included in the present investigation did not blind the patients to the treatment allocation. However, a blinded allocation is hardly possible in the comparison of APM and PT and it must be pointed out by us. The RCTs which evaluated the efficacy of sham arthroscopy versus APM were conducted in a double blinded fashion. Assessors were often not blinded to the patient treatment, increasing the detection bias. In some RCTs, many patients allocated to PT crossed over, undergoing APM before assessment of the primary outcome. Moreover, some inconsistencies in VAS score must be pointed out. Some authors did not report whether VAS referred to the pain at rest, during sports, or during daily activities. The location, type, and degree of the lesions in the meniscus were often biased; therefore, it was not possible to evaluate the efficacy of APM in these subgroups. In this respect, the reliability of the conclusion of the present systematic review are not fully generalisable. Future studies should evaluate the efficacy and safety of APM for each specific place, type, and degree of meniscal lesion. The presence of chondral defects was seldom considered for patient eligibility. Chondral defects are common, especially in middle aged adults [17, 48]. Given the limited healing potential of hyaline cartilage, these lesions are most likely unable to regenerate [16, 37]. If left untreated, patients with chondral defects may experience chronic instability, pain, and early onset osteoarthritis, along with significant reduction in the quality of life and participation to recreational activities [36, 39]. Most studies investigated PROMs and imaging findings to assess knee degeneration. However, no other imaging methodology has been used to verify whether additional modifications to the meniscus occurred at the end of the follow-up. The description of surgical technique was not adequately reported in some studies, representing a further limitation. Moreover, the included studies referred to different PROMs, which produce high variability in the endpoints. Given the lack of available pooling data, along with the heterogeneous PROMs used by the authors, further subgroup analyses were not possible. Moreover, the use of PROMs was inadequate in most studies. PROMs which focus on meniscus (i.e., Western Ontario Meniscal Evaluation Tool, WOMET) have been used only in one study. The degree of OA according to the Kellgren–Lawrence scale was slightly variable; however, no study included patients with Kellgren–Lawrence IV. Similar considerations apply for the age of the patients. The rage of patients age was wide (from 35 to 70 years), which may lead to increase the selection bias.

Further long-term RCTs are required to investigate whether APM and PT produces comparable results using validated and reliable PROMs. Moreover, future RCTs should investigate whether patients who might benefit from APM actually exist, clarifying proper indications and outcomes. In the current literature, little is published on prognosticators of the outcome of PT and on results of crossover to surgery for meniscal damage. Although rehabilitation is important for meniscus healing in meniscus ailments or following arthroscopy, the optimal rehabilitation regimen has also not been established. These issues should be addressed by future investigations.

## Conclusions

The benefits of APM in adults with degenerative and nonobstructive meniscal symptoms are limited. The current evidence reports similarity in the outcome between APM and PT. Further long-term RCTs are required to investigate whether APM and PT produces comparable results using validated and reliable PROMs. Moreover, future RCTs should investigate whether patients who might benefit from APM exist, clarifying proper indications and outcomes. High quality investigations are strongly required to establish the optimal PT regimes.

## Data Availability

the data sets generated during and/or analysed during the current study are available from the corresponding author on reasonable request.
